# Absence of a secondary glucocorticoid response in C57BL/6J mice treated with topical dexamethasone

**DOI:** 10.1371/journal.pone.0192665

**Published:** 2018-03-02

**Authors:** Jennifer A. Faralli, Kaylee D. Dimeo, Ralph M. Trane, Donna Peters

**Affiliations:** 1 Department of Pathology & Laboratory Medicine, University of Wisconsin School of Medicine and Public Health, Madison, Wisconsin, United States of America; 2 Department of Ophthalmology & Visual Sciences, University of Wisconsin School of Medicine and Public Health, Madison, Wisconsin, United States of America; Oregon Health and Science University, UNITED STATES

## Abstract

Glucocorticoids such as dexamethasone can cause an increase in intraocular pressure (IOP) in some of the population, but not all. In this paper we used a mouse model of glucocorticoid induced ocular hypertension to examine the changes in the anterior segment of the eye in mice that failed to respond to glucocorticoid treatment with a sustained increase in IOP. C57BL/6J mice were treated with either 0.1% dexamethasone sodium phosphate ophthalmic solution or sterile PBS 3 times daily for up to 5 weeks. IOP was measured weekly at approximately the same time of the day. After 3–5 weeks of treatment, eyes were enucleated and evaluated for changes associated with steroid induced glaucoma. These studies showed that IOP was significantly elevated in dexamethasone (DEX) treated mice compared to PBS treated mice after 3 weeks of treatment, but IOP in DEX treated mice returned to baseline levels after 5 weeks of treatment. All the mice demonstrated a response to the glucocorticoid treatments and showed an elevation in FKBP5 expression after both 3 and 5 weeks of DEX treatment (primary glucocorticoid response protein) and a weight loss. Western blot analysis of anterior segments from treated mice, however, did not show an increase in secondary glucocorticoid response proteins such as β3 integrin or myocilin. Fibronectin levels were also not statistically different. The data suggest that in mice, which do not exhibit a prolonged increase in IOP in response to the DEX treatment, there is a compensatory mechanism that can prevent or turn off the secondary glucocorticoid response.

## Introduction

Glaucoma is a heterogeneous disease manifested by progressive degeneration of the optic nerve head that eventually leads to permanent blindness. A major risk factor for glaucoma is increased intraocular pressure (IOP). About 70 million people are affected by the most common form of glaucoma called primary open angle glaucoma (POAG) [1, 2]. Another form of glaucoma called steroid induced glaucoma (SIG) results from long-term use of glucocorticoids such as dexamethasone (DEX). The development of SIG is thought to be similar to POAG since over 90% of POAG patients respond to topical steroid treatment with increased IOP. In addition, both POAG and SIG show similar morphological and functional changes in the trabecular meshwork (TM) and both exhibit an increase in IOP that results from a restriction of aqueous humor through the TM [3–5]. However, only about 30%-40% of the normal population develop elevated IOP when treated with glucocorticoids for 4–6 weeks [4, 6–9] and after removal of the steroid treatment, IOP returns to baseline for most patients. However, 4–6% of the patients that respond to steroids with a large increase in IOP are considered to be high responders and are more likely to develop POAG [4, 10]. Additionally, in 1–3% of the patients who respond to steroids, IOP remains elevated after the withdrawal of the glucocorticoid and the patients go on to develop SIG. [11–13]

The molecular mechanisms that cause POAG and SIG are not clear. Nor is it clear why some individuals respond to glucocorticoids with an elevation in IOP and others do not. Studies using C57BL/6J mice treated systemically or topically with DEX showed an elevation in IOP and ultrastructural changes similar to those reported in humans following treatments with glucocorticoids [14, 15]. In particular, the DEX treated mice showed an upregulation of fibronectin (FN), myocilin (MYOC) and the transcription factor CHOP (CCAAT-enhancer-binding protein homologous protein) which is a marker for chronic ER stress. Importantly, not all mice exhibit an increase in IOP following treatment with a glucocorticoid [16, 17], suggesting that similar to humans, not all mice are steroid responders. In vitro studies using human TM cells have indicated that lower levels of the alternatively spliced glucocorticoid receptor β may be responsible for the initial responsiveness to glucocorticoids that lead to the altered expression of extracellular matrix proteins upregulated in POAG [18].

Interestingly, many of the changes associated with steroid induced increases in IOP are the result of the activation of a secondary glucocorticoid response. The secondary glucocorticoid response differs from a primary glucocorticoid response in two important ways. First, genes upregulated by this mechanism lack classical glucocorticoid response elements (GRE) and thus the glucocorticoid receptor does not directly interact with these genes. Rather, the glucocorticoid receptor triggers the de novo protein synthesis of another soluble factor, which in turn is responsible for the transcription of the gene. Second, it takes hours to days to see increased gene expression following steroid exposure rather than minutes as observed in a primary glucocorticoid response.

Two proteins found in cultures of TM cells to be the result of a secondary glucocorticoid response [19–22] are MYOC and the β3 integrin subunit, both of which lack classic GREs. Fibronectin is another protein frequently shown to be upregulated by glucocorticoids in TM cell cultures that also lacks GREs and may be considered a secondary glucocorticoid response [23]. Recent studies have shown that both MYOC and αvβ3 integrin expression is dependent on the activation and nuclear translocation of the transcription factor NFATc1 [19, 24]. Mutations in MYOC are responsible for some forms of POAG and juvenile open-angle glaucoma [25] whereas the upregulation and activation of the αvβ3 integrin [24, 26] has recently been shown to be involved in many of the phenotypic changes associated with SIG and POAG [26–28]. For instance, activation of the αvβ3 integrin signaling pathway in TM cells regulates several cytoskeletal events thought to be significant in glaucoma including decreased phagocytosis [29, 30] and the organization of actin filaments into cross-linked actin networks called CLANs [26–28]. CLANs are believed to modulate the contractile properties of TM cells by making them rigid and unable to respond to changes in eye pressure [31]. They are found in increased numbers in glaucomatous and DEX-treated tissues [32] and in DEX-treated TM cell cultures [26, 33].

In this study, we used the DEX mouse model of ocular hypertension to examine the secondary glucocorticoid response in vivo. Anterior segments from mice who responded to DEX treatments but failed to show a prolonged elevation in IOP were analyzed for the expression of proteins associated with the secondary glucocorticoid response. These studies show that the secondary glucocorticoid response is absent in these mice that do not exhibit a sustained increase in IOP. Understanding what happens when steroid responders are able to prevent an elevation in IOP should enhance our understanding of the steroid-induced mechanism(s) involved in POAG and SIG.

## Materials and methods

### Animals

Male C57BL/6J mice were obtained from the Jackson Laboratory and were housed at the University of Wisconsin animal facilities. The mice were housed in a 12-hour light/12-hour dark cycle with food and water freely available. All animal studies were carried out in accordance with the Association for Research in Vision and Ophthalmology Statement for the Use of Animals in Ophthalmic and Vision Research and were approved by the Institutional Animal Care and Use Committee of the University of Wisconsin-Madison School of Medicine and Public Health (protocol # M005242).

### Intraocular pressure measurements and dexamethasone treatment of mice

Male mice (8–16 weeks of age) were anesthetized intraperitoneally with a ketamine/xylazine mix (90mg/10mg per kg) and IOP was measured as soon as the mice stopped moving before the anesthesia effect on IOP occurred using an Icare Tonolab. Three IOP measurements from each eye were averaged together at each time point. After baseline IOP was measured, the right eye was treated topically with one drop (~30μl) of either 0.1% DEX sodium phosphate ophthalmic solution (Bausch & Lomb) or sterile PBS 3 times daily, 4 hours apart, for up to 5 weeks. The contralateral eyes were left untreated. IOP was measured weekly at approximately the same time of the day under light conditions. This procedure was repeated six times using 8 or 16 mice in each group.

### Tissue processing

After 3–5 weeks of DEX or PBS treatment, mice were euthanized with carbon dioxide and eyes were enucleated and processed one of three ways. Some eyes were bisected just posterior to the limbus and the anterior segments were put into RNA Later (Invitrogen) and frozen for subsequent RNA isolation. Other eyes were bisected and the anterior segments were lysed for western blotting. Lastly, some anterior segments were fixed in 4% paraformaldehyde and paraffin embedded for immunohistochemistry.

### RNA isolation and RT-PCR

Anterior segments were processed as individual eyes. Total RNA was isolated using the QIAshreddar and RNeasy Plus Mini Kits (Qiagen, Inc.) according to the manufacturer’s instructions after homogenizing the anterior segments (including scleral, cornea, iris, ciliary muscle and TM) with a disposable micropestle in 350μl of lysis buffer provided by the kit. RNA concentration was determined using a NanoDrop spectrophotometer. RT-PCR was performed using the Superscript SYBR Green 1-step RT-PCR kit according to the manufacturer’s instructions (Thermo Fisher Scientific). Fold changes in gene expression were determined using the method described by Pfaffl [34] that corrects for primer efficiency. Data were normalized to no treatment and fold change compared to the housekeeping gene succinate dehydrogenase complex subunit A (SDHA) was determined. Primers used are in Table 1.

**Table 1:** Primers used for RT-PCR.

Gene	Forward primer (5’ to 3’)	Reverse primer (5’ to 3’)
*SDHA*	GGAACACTCCAAAAACAGACCT	CCACCACTGGGTATTGAGTAGAA
*Itgb3* (β3 integrin)	GCTCATTGGCCTTGCTACTC	GGTGGAGGTGGCCTCTTTAT
*FKBP5*	GATGAGGGCACCAGTAACAATG	CAACATCCCTTTGTAGTGGACAT
*MYOC*	GACAGCACAGTTCCGAAAGG	GGGCAGCTAGATTCATTGGGG
*DDIT3* (CHOP)	ACAGAGGTCACACGCACATC	GGGCACTGACCACTCTGTTT

### Western blotting

Anterior segments were immediately placed in 200μl ice cold lysis buffer after dissection (25mM Hepes, pH 7.4, 150mM NaCl, 1mM EDTA, 1mM NaF, 1% NP-40, 0.25% DOC, and Halt protease and phosphatase inhibitor cocktails (Thermo Fisher Scientific)). Tissue was then sonicated (Branson Sonifier SLPe) 5 times for 1 second using 30% amplitude and insoluble material was removed by centrifugation at 14,000 rpm for 10 min at 4°C. A BCA assay (Thermo Scientific Pierce Micro BCA Protein Assay Kit) was performed on the supernatant to determine protein concentration. Lysate (10μg) was separated on a 10% SDS-PAGE (6% for FN) and transferred to Immobilon-FL (Millipore Corp). The membrane was blocked with 3% bovine serum albumin (BSA) or 5% milk (for FKBP5 antibody) in Tris buffered saline with 0.5% Tween-20 (TBST) for at least 1h at room temperature. Membranes were then incubated overnight at 4°C with primary antibody diluted in 1% BSA or 5% milk (FKBP5) in TBST. After washing, membranes were incubated for 1h at room temperature with IR Dye 800 conjugated goat anti-mouse or anti-rabbit secondary antibody (Licor) diluted 1:15,000 in 1% BSA or 5% milk (FKBP5) in TBST with 0.01% SDS. Membranes were washed and then scanned using the Odyssey CLx imager (Licor). Antibodies used include mouse anti-β3 integrin clone AP-3 (1:1000; Kerafast), rabbit anti-MYOC (1:1000; Abcam ab41552), rabbit anti-FKBP5 (1:1000; Cell Signaling Technologies 12210S), rabbit anti- (1:1000 [35]), rabbit anti-CHOP (1:500; Abcam ab179823), and rabbit anti-β-actin (1:1000, Abcam ab8227). Densitometry of the bands was determined using Image Studio v. 5.0 software (Licor).

### Immunohistochemistry

Anterior segments were fixed in 4% paraformaldehyde in PBS for 1 h then transferred to PBS until embedded in paraffin. Sections (5μm thick) were deparaffinnized in xylene and rehydrated through a series of 100–50% ethanol solutions followed by immersion in water. Antigen retrieval was performed on the sections by incubated in 1 mM EDTA, pH 8.0 for at 95°C for 20 min. Once cooled, sections were blocked at least 2 h in 3% BSA in PBS at room temperature. Sections were then incubated overnight at 4°C with rabbit anti-β3 integrin antibody (Abcam ab197662) at 1:50. After washing 5 times with PBS, sections were incubated for 1 h at room temperature with Alexa 546 conjugated goat anti-rabbit secondary antibody (Invitrogen) at 1:500. Sections were then stained with Hoescht 33342 (Invitrogen) in PBS to label nuclei and then mounted with Immu-mount (Thermo Scientific Shandon). Images were acquired with a Nikon A1Rs confocal microscope using NIS Elements software at the University of Wisconsin Optical Imaging Core.

### Data analysis

Data are presented as mean ± S.E.M. Statistical comparisons were done using the Student unpaired t-test and a p value <0.05 was considered significant. For RT-PCR analysis, statistical comparisons were done on the log scale using the Student unpaired t-test and a p value <0.05 was considered significant. Means with 95% confidence intervals were then transformed back to the original scale on which they are represented.

## Results

**Fig 1:**
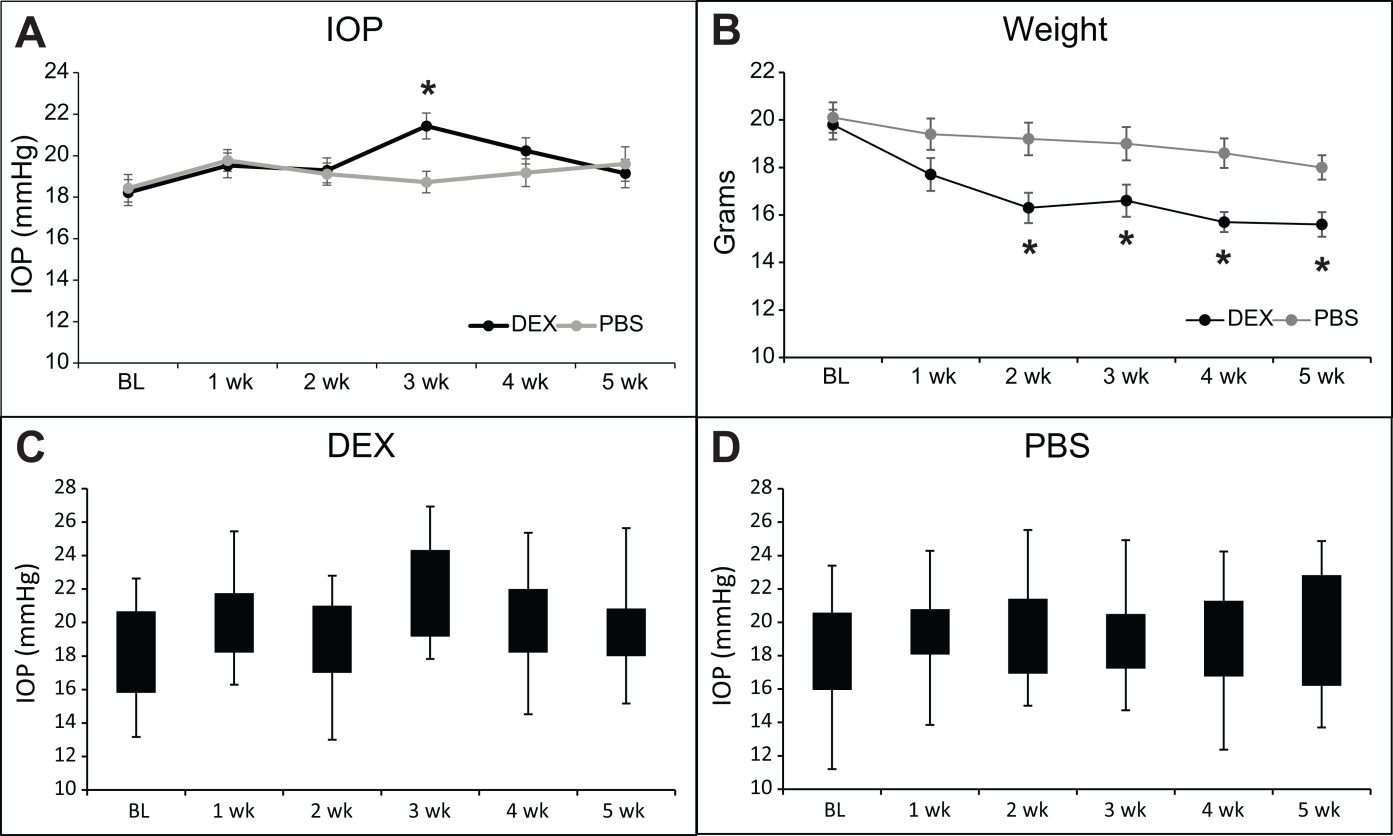
DEX treatment transiently increases IOP in mice. (A) Topical DEX or PBS was administered 3 times a day for 4 to 5 weeks (n = 17–21 for DEX and n = 20–24 for PBS; see Table 2). Graph shows average IOP. IOP of DEX treated eyes is significantly different than PBS treated eyes, *p<0.05. (B) Average weight of mice over the course of treatment. Weight is significantly different than at baseline (BL), *p<0.05. (C) Box and Whisker plot of IOP data from DEX treated mice showing IOP distribution. (D) Box and Whisker plot of IOP data from PBS treated mice showing IOP distribution.

**Fig 2:**
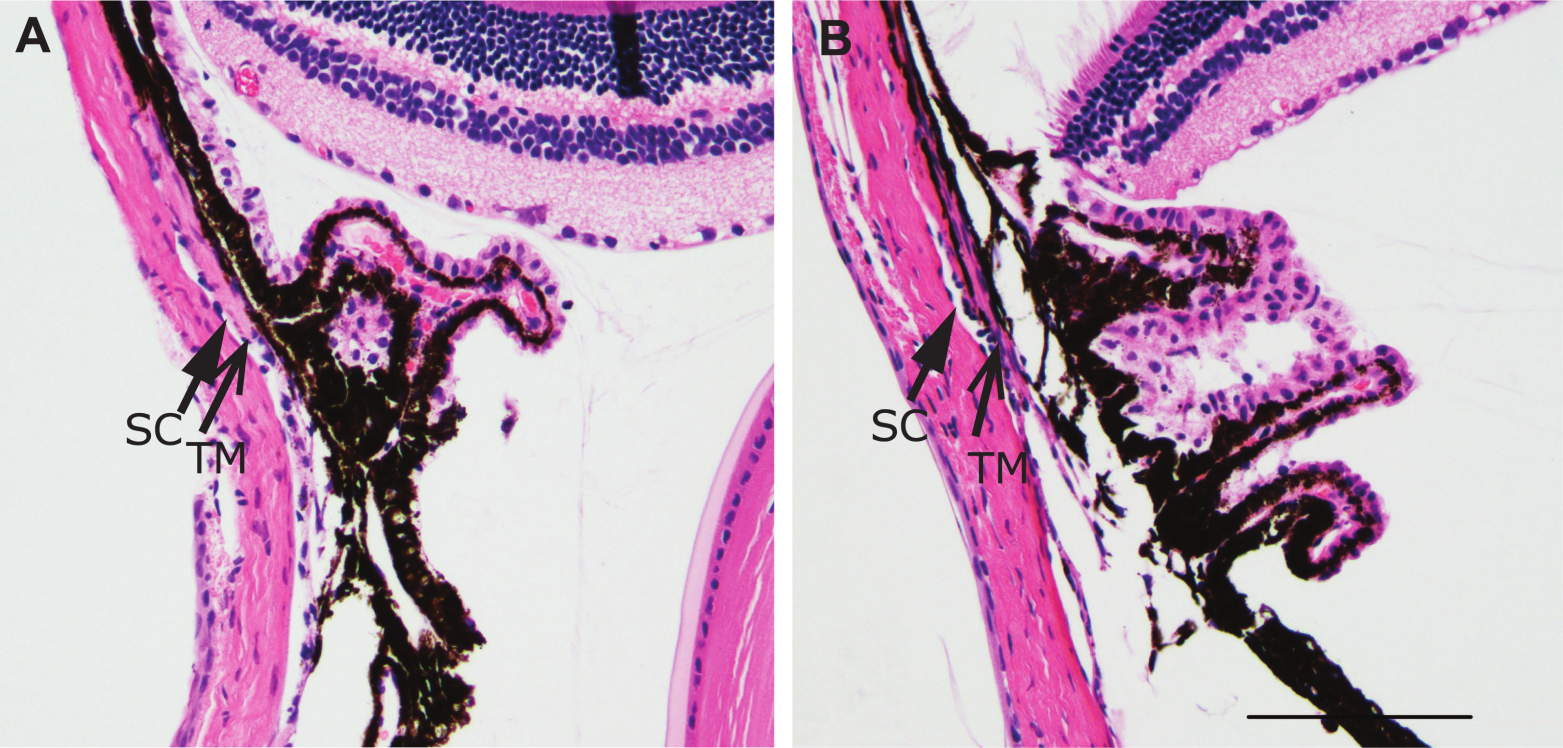
Hematoxylin and eosin (H&E) stain of paraffin sections. Representative H&Es of the chamber angle of eyes treated for 5 weeks with DEX (A) or PBS (B). Magnification bar = 100μm.

A total of 48 mice were treated with DEX or PBS for 4 or 5 weeks. During the study, two mice treated with DEX died due to causes unrelated to the study. One DEX treated mouse responded to the DEX treatment with a large increase in IOP. This mouse was excluded in the final analysis since we are studying mice who do not respond to glucocorticoids with a sustained increase in IOP. As shown in Fig 1A, IOP increased after 3 weeks of DEX treatment compared to PBS treatment, but then returned to baseline by 5 weeks of DEX treatment. At 3 weeks of treatment, the average IOP of DEX treated eyes was statistically significantly higher than the average IOP of PBS treated eyes (Fig 1A; 21.4+/- 0.6 vs. 18.7+/- 0.5 mmHg; p<0.05). This elevation of 2.7mmHg, although mild, is very similar to what was seen by others following treatment with a glucocorticoid [14, 16]. Out of the 21 mice treated with DEX, 17 mice (81%) showed at least a 2 mmHg increase (2 to 8.1mmHg) in their IOP relative to baseline and 4 showed less than a 2 mmHg increase or a decrease in IOP (-3.2 to 1.7mmHg) after 3 weeks of the treatment (Table 2). In contrast, out of the 24 mice treated with PBS, only 7 mice (29%) in the PBS treatment group showed an increase of at least 2 mmHg in IOP (2 to 7.3mmHg) compared to baseline while 17 mice showed near baseline IOP or a decrease in IOP (-5.6 to 1.2mmHg) compared to baseline. By 4 weeks the IOP of DEX treated mice had decreased and was no longer significantly different compared to PBS treated mice (20.2+/- 0.6 vs. 19.2+/- 0.7 mmHg) and by 5 weeks the groups were the same (19.1+/-0.7 vs. 19.6+/- 0.8 mmHg). We also looked at the IOP differences of individual mice at 5 weeks of treatment to determine if any DEX treated mice still exhibited increased IOP, i.e. steroid responders. Of the eyes treated with DEX or PBS for 5 weeks, 5 of the 17 (29%; 2.2 to 6.6mmHg) DEX treated eyes and 5 of the 20 (25%; 2.2 to 6.2mmHg) PBS treated eyes were at least 2 mmHg higher than baseline. Because these percentages are similar it suggests that none of the mice in our DEX study developed a prolonged elevation in IOP. Fig 1C and 1D show the range of the IOPs for each week of treatment. We also compared IOPs to baseline IOP for each treatment. DEX treated eyes exhibited higher IOPs compared to baseline IOP of the same eyes after 3 weeks (21.4+/-0.6 vs. 18.2+/-0.6 mmHg; p<0.05) and 4 weeks (20.2+/-0.6 vs. 18.2+/-0.6 mmHg; p<0.05) of treatment while PBS treated eyes were no different from baseline. Thus in our experiments, IOP was not increased in mice treated for 5 weeks with topical DEX eye drops. The mice treated with DEX, however, showed a steady and significant decrease in their weight over the 5 week period that was greater than the weight loss in the PBS treated mice (Fig 1B) indicating that systemic effects with the DEX treatment [14, 16] resulting from either absorption through the nasalacrimal mucosa or through ingestion from grooming had occurred. This systemic absorption or contralateral effect has been observed in mice with atropine [36, 37] and clinically in humans following topical application of beta-blockers or timolol to reduce IOP [38, 39] Immunohistochemical studies indicated that the anterior segments of mice treated with DEX appeared similar to PBS treated mice and did not show any gross morphological differences despite the 5 week treatment with DEX (Fig 2).

**Table 2:** Individual mouse IOPs.

**DEX treated eyes**		**BL**	**1 week**	**2 weeks**	**3 weeks**	**4 weeks**	**5 weeks**
Group 1	M7	14.7	14.5	16	22.8	16.7	20.8
	M8	13.8	18.7	22.3	18.3	21.7	15.5
	M9	14	17.6	21.3	16.6	15.7	15.3
Group 2	M15	15.7	19.5	19.8	19.5	17	
	M16	17.3	14.6	20	21.2	14.8	
	M17	18.3	20	17.5	22.7	19	
	M18	17.5	19.7	17.8	19.2	19.3	
Group 3	M31	17.8	20.2	17.6	19.8	21.8	20
	M32	15.8	18.4	17	18.4	18.2	19.8
	M33	19.8	17	16.8	16.6	19.6	19
Group 4	M39	17.2	18.4	15.4	24.6	19.4	15.4
	M40	17.4	18.2	15.2	25.4	19.4	13.2
	M42	14.4	22	18.8	19	19.6	21
Group 5	M453	19.0	22.3	20.3	21.3	23.3	20.7
	M454	23.3	21.8	21	25.7	22	20.7
	M455	22.7	23.7	22.7	24.7	24.8	21.8
	M456	22	15.3	16.3	23	18	18
	M457	21	23.7	20.3	24.3	19.7	23.7
	M458	20.7	22	23.3	22.3	25.7	22.3
	M459	19.7	21.3	25	24.5	23.8	19
	M460	20.7	21.3	20.7	20.3	23	19.3
**PBS treated eyes**		**BL**	**1 week**	**2 weeks**	**3 weeks**	**4 weeks**	**5 weeks**
Group 1	M11	18.3	14.6	16.7	19.5	16	17.3
	M12	13.1	18.1	20.7	18	19.5	14.2
	M13	14.9	20.2	21.8	14.3	21.1	15.5
	M14	15.3	17.7	17.5	19.5	16.3	21.5
Group 2	M19	16	18.7	22.8	18.2	18.8	
	M20	15.7	21.2	22.2	23	20.3	
	M21	16	17.8	18.5	19.8	18.7	
	M22	17.5	19.5	18.8	17.5	16.8	
Group 3	M35	17.4	17.6	17	16.4	13.8	15.2
	M36	18.4	18.6	18.6	12.8	15.6	20.6
	M37	18.8	17	19	18.4	16.8	19.4
	M38	15.4	19.4	16.4	19.4	16.6	20.8
Group 4	M43	17.2	19.2	12.8	16	19.4	17.8
	M44	16.2	18.4	16	16	17.8	15.8
	M45	15.8	20.6	16.4	18	17	16.6
	M46	18	17.2	16.6	15.8	15.2	15.4
Group 5	M449	22.3	25	20.8	22	20	25
	M450	25.3	22.3	19.3	19.7	25	20.7
	M451	24.7	23	22	21	25.3	16.3
	M452	21.3	25	19.7	21	21.7	23.3
	M461	22	19.7	21.3	20.3	25.7	25.3
	M462	20.3	23.8	23.3	21	22	24.3
	M463	19.7	20.7	19	19.7	18.7	22.7
	M464	22.7	19.3	21.7	22.3	22.7	24.3

**Fig 3:**
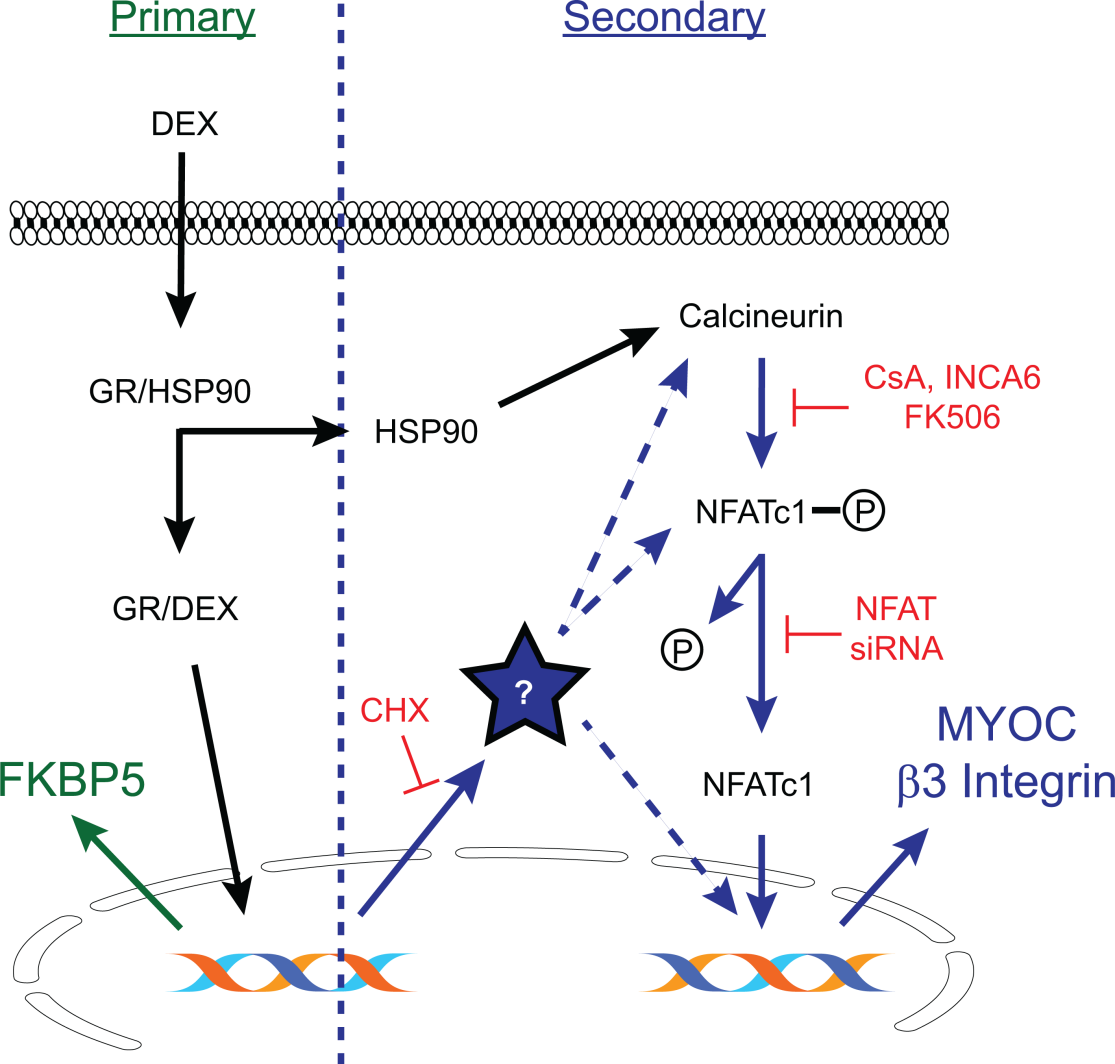
Primary vs. secondary glucocorticoid response pathways. A primary glucocorticoid response pathway (shown in green) occurs when exposure to glucocorticoids such as DEX causes activation of the glucocorticoid receptor (GR) in the cytoplasm. The GR/DEX complex then translocates to the nucleus and within minutes activates gene transcription of genes that contain glucocorticoid response elements. FKBP5 is an example of a primary glucocorticoid response gene. During a secondary glucocorticoid response (shown in blue), the GR/DEX complex induces transcription followed by translation of some soluble factor (?) that in turn is needed to activate transcription of a second gene. This process often takes hours to days to occur and can be inhibited by cycloheximide (CHX). This model shows the activation of the secondary glucocorticoid response genes myocilin (MYOC) and β3 integrin by the transcription factor NFATc1 [19, 24]. It is unknown how the soluble factor induced by the GR/DEX complex activates the transcription factor NFATc1. It could act directly, through the phosphatase calcineurin which in turn activates NFATc1 or it could be part of the complex responsible for the nuclear translocation of NFATc1. Alternatively, it could interact with NFATc1 to co-regulate transcription of secondary glucocorticoid response genes.

**Fig 4:**
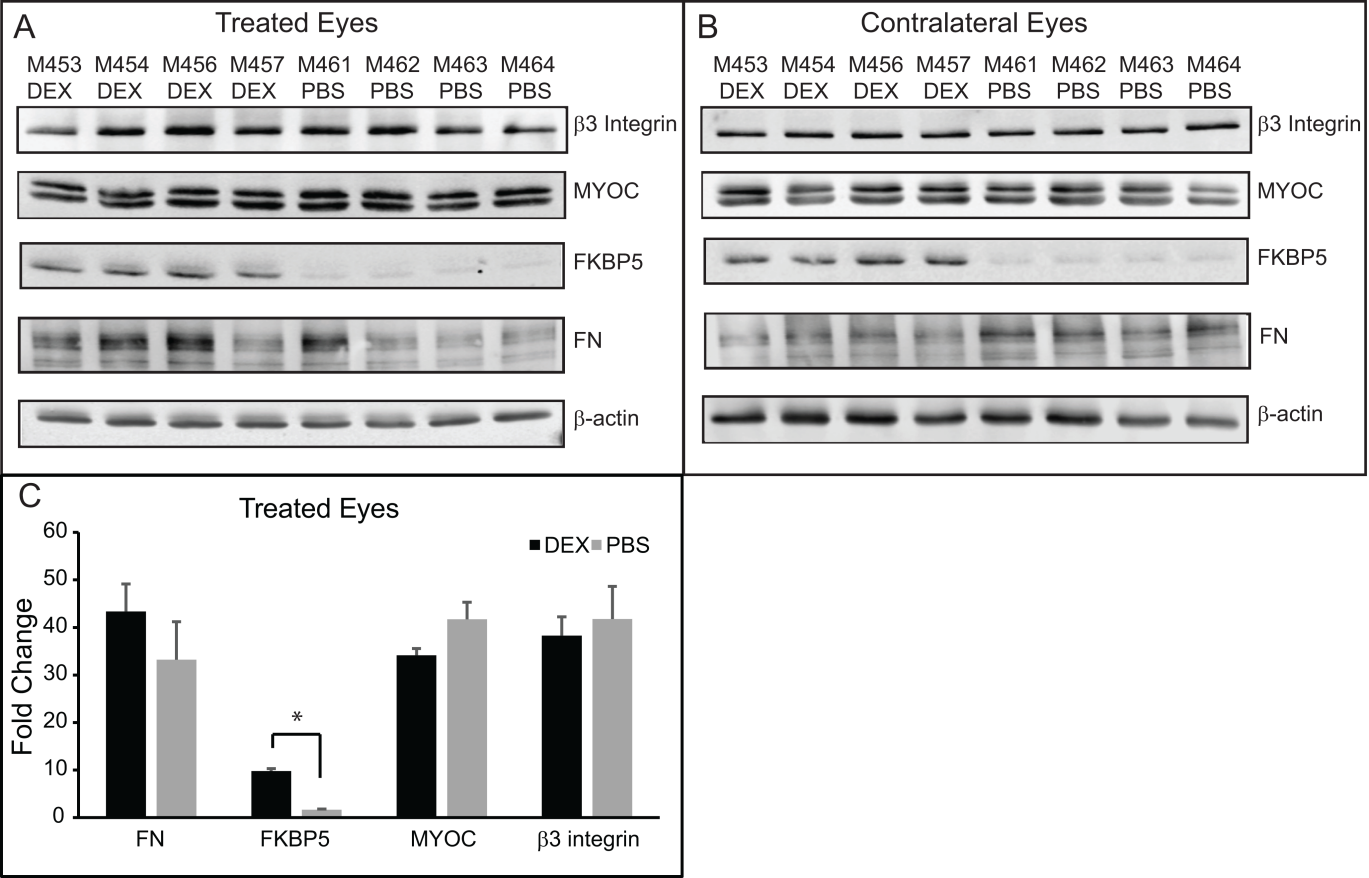
Western blotting of lysates from anterior segments of mouse eyes treated with DEX or PBS for 5 weeks. (A) Western blots of lysates from eyes treated with DEX or PBS for 5 weeks. (B) Western blot of lysates from the contralateral untreated eyes of the same mice as in (A). (C) Densitometry of western blots shown in (A), normalized to the β-actin loading control. DEX treated versus PBS treated eyes were significantly different, *p<0.05.

We then performed western blotting analysis to determine if proteins known to be upregulated by DEX treatment in human TM cell cultures established from anterior segments [19, 24, 40] were also upregulated in the anterior segments from mice treated with topical DEX. We first looked to see if FKBP5 was upregulated. FKBP5 is known to contain glucocorticoid response elements (GREs) and upregulation of its expression would demonstrate that the primary glucocorticoid response was activated (Fig 3). As shown in Fig 4A and quantitated in Fig 4C, FKBP5 was upregulated in DEX treated eyes versus PBS treated eyes as determined by densitometry (9.8 +/- 0.5 vs. 1.6 +/-0.2; p<0.05). FKBP5 was also upregulated in the contralateral untreated eyes of the DEX treated mice (Fig 4B), supporting the fact that there were systemic effects from the topical DEX treatments.

We then looked for proteins associated with the secondary glucocorticoid response in human TM cells (Fig 3). As shown in Fig 4A and 4C, there was no difference in β3 integrin or MYOC expression between DEX treated eyes compared to PBS treated eyes. We also looked at FN expression whose increased expression levels may also result from a secondary glucocorticoid response like MYOC and β3 integrin, since it does not contain any GREs [23]. As shown in Fig 4A, there was a trend of higher FN expression in DEX treated eyes compared to PBS treated eyes, but this was not statistically significantly different by densitometry (Fig 4C). Finally, we looked for an increase in CHOP which has also been reported to be increased in the anterior segments of mice treated with topical DEX drops [15]. However, we were unable to detect CHOP expression in the DEX or PBS treated eyes (S1 Fig). These data suggest that the secondary glucocorticoid response was not activated in the DEX treated mice after 5 weeks of treatment.

**Fig 5:**
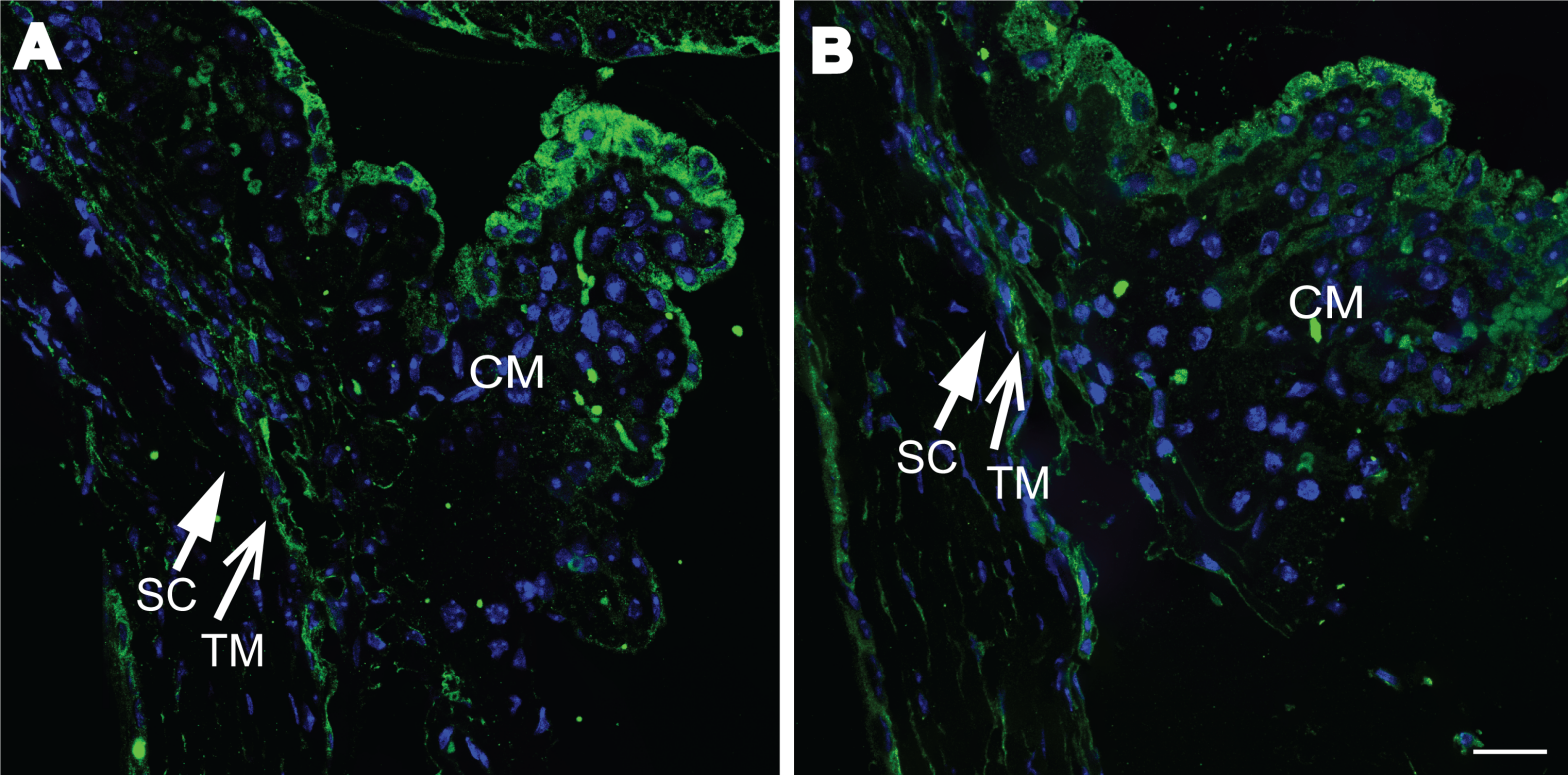
β3 integrin labeling of paraffin sections. Representative β3 integrin labeling (green) of paraffin sections from eyes treated for 5 weeks with DEX (A) or PBS (B). Sections were stained with Hoechst 33342 (blue) to view nuclei. Schlemm’s canal (SC) is indicated with a closed arrow. TM is indicated with an open arrow. Magnification bar = 10μm.

We then labeled sections of paraffin embedded eyes treated with DEX or PBS for β3 integrin and MYOC. Fig 5A and 5B show no obvious differences in β3 integrin levels in the TM between DEX and PBS treated eyes. We were unable to detect any MYOC expression using two different antibodies, despite being able to detect MYOC by western blotting (S2 Fig). It is unclear if that is because the antibodies used did not work for this application. Together these data suggest that the secondary glucocorticoid response in the TM of DEX treated mice was not activated or was downregulated after 5 weeks of treatment.

**Fig 6:**
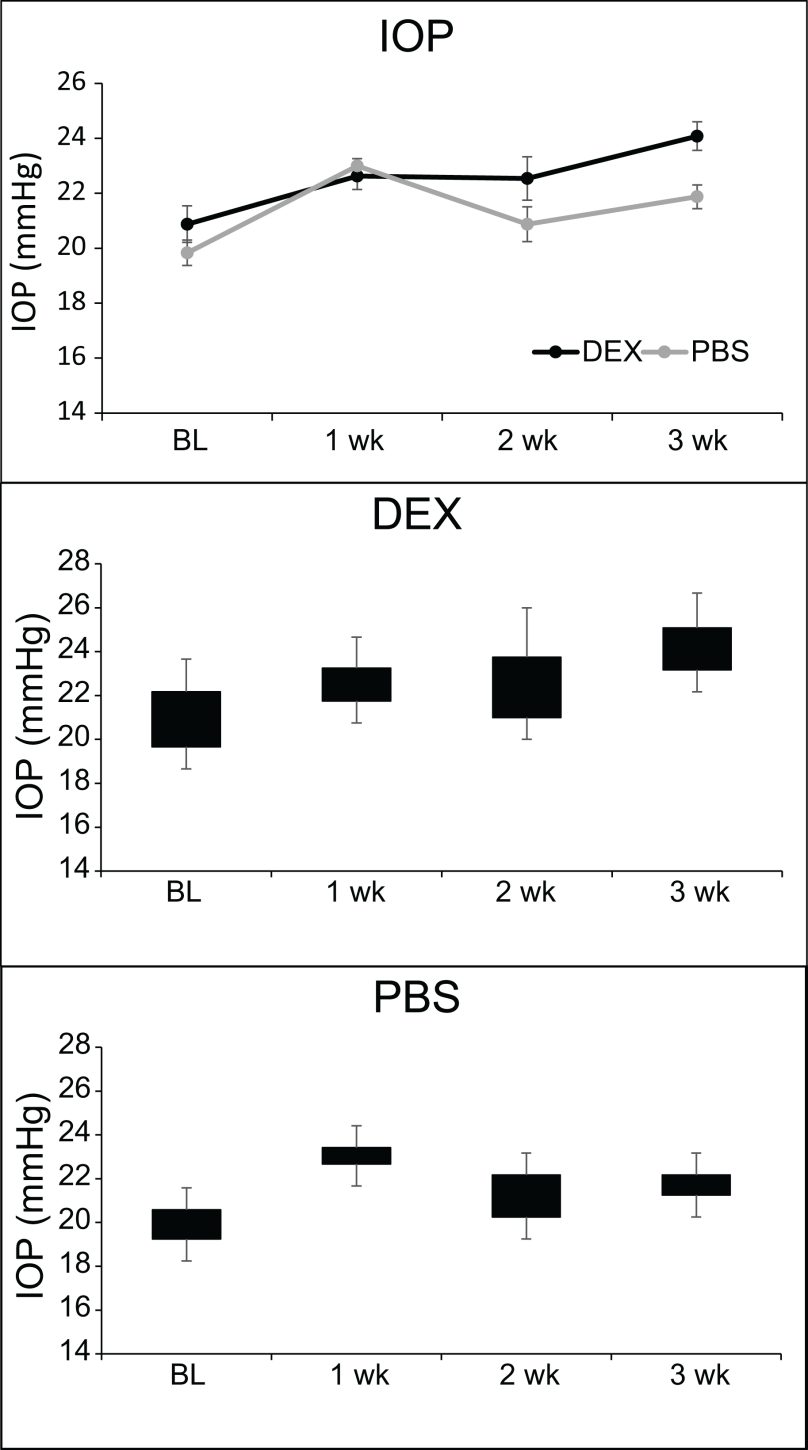
DEX treatment increases IOP in mice. (A) Topical DEX or PBS was administered 3 times a day for 3 weeks (n = 8 for DEX and n = 8 for PBS). Graph shows average IOP. IOP of DEX treated eyes is significantly different than PBS treated eyes, *p<0.05. (B) Box and Whisker plot of IOP data from DEX treated mice showing IOP distribution. (C) Box and Whisker plot of IOP data from PBS treated mice showing IOP distribution.

**Fig 7:**
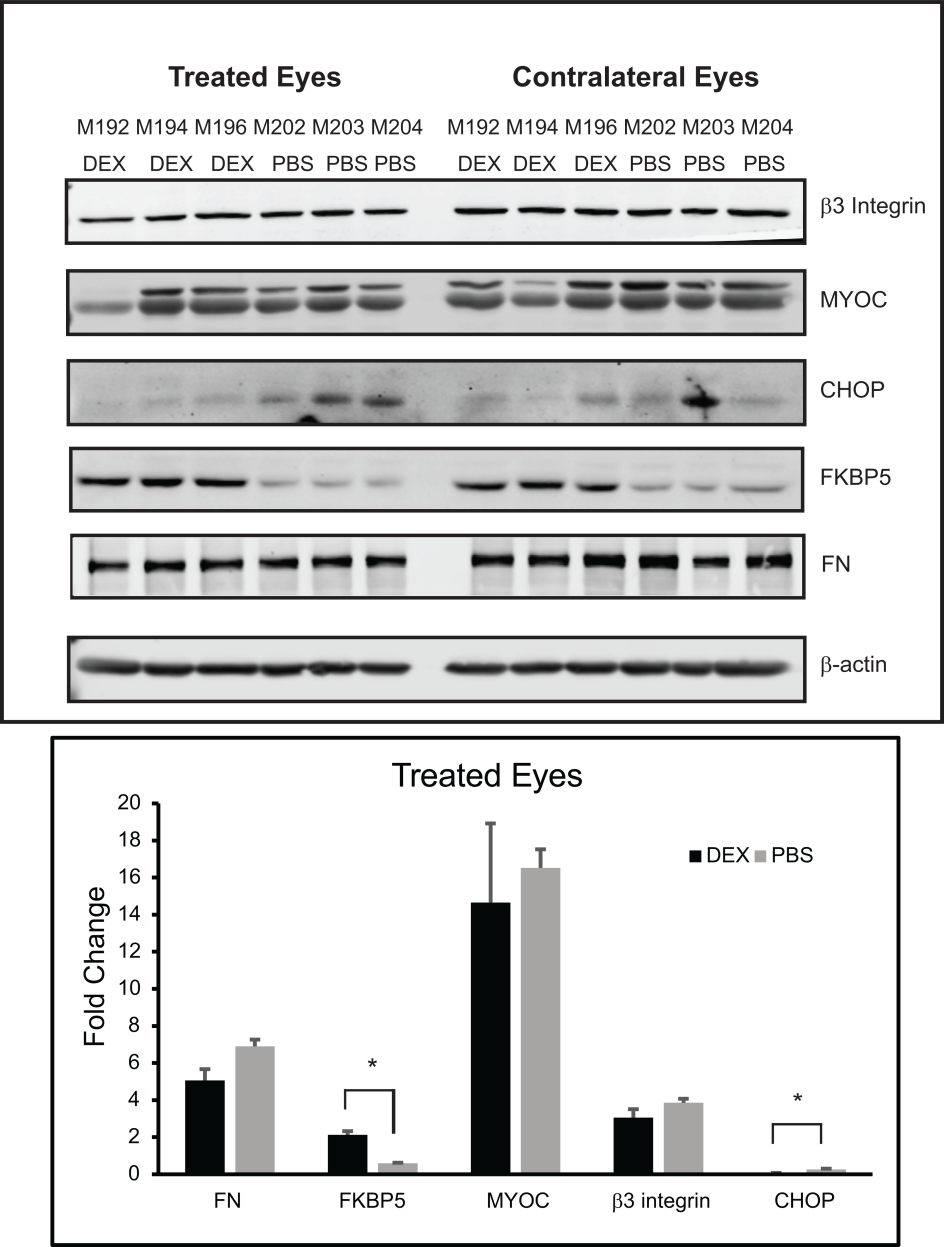
Western blotting of lysates from anterior segments of mice eyes treated with DEX or PBS for 3 weeks. (A) Western blots of lysates from eyes treated with DEX or PBS and the contralateral untreated eyes of the same mice. (B) Densitometry of western blots shown in (A), normalized to the β-actin loading control. DEX treated versus PBS treated eyes were significantly different, *p<0.05.

To get a better understanding of why an elevation in IOP was not maintained at 5 weeks despite the response to the DEX treatment and initial elevation in IOP, we repeated the study so we could examine the tissues after 3 weeks of DEX treatment when the IOP was increased compared to the control group of mice. Fig 6A shows the average IOP of mice treated in this group. As before we saw an increase in IOP by 3 weeks of treatment with DEX. The average IOP of DEX treated eyes compared to PBS treated eyes was statistically significantly greater at 3 weeks (24.0+/-0.5 vs. 21.9+/-0.4 mmHg; p<0.05). Fig 6B and 6C show the range of the IOPs for each week of treatment. Anterior segments of mice from each group were then evaluated by western blotting for the same proteins as the 5 weeks of treatment shown above. As shown in Fig 7A, there was no change in β3 integrin, MYOC or FN levels between DEX treated versus PBS treated mice eyes, similar to the 5 week data. Like the 5 week data, FKBP5 was significantly upregulated in the DEX treated eyes as well as the contralateral eyes from the same mice compared to the eyes from PBS treated mice. The expression of CHOP, however, was different from the 5 week samples and we were able to detect CHOP in lysates from both the DEX and PBS 3 week treated samples. Interestingly, densitometry of the western blot showed a significant decrease in the amount of CHOP present in DEX treated eyes compared to PBS treated eyes (p<0.05).

**Fig 8:**
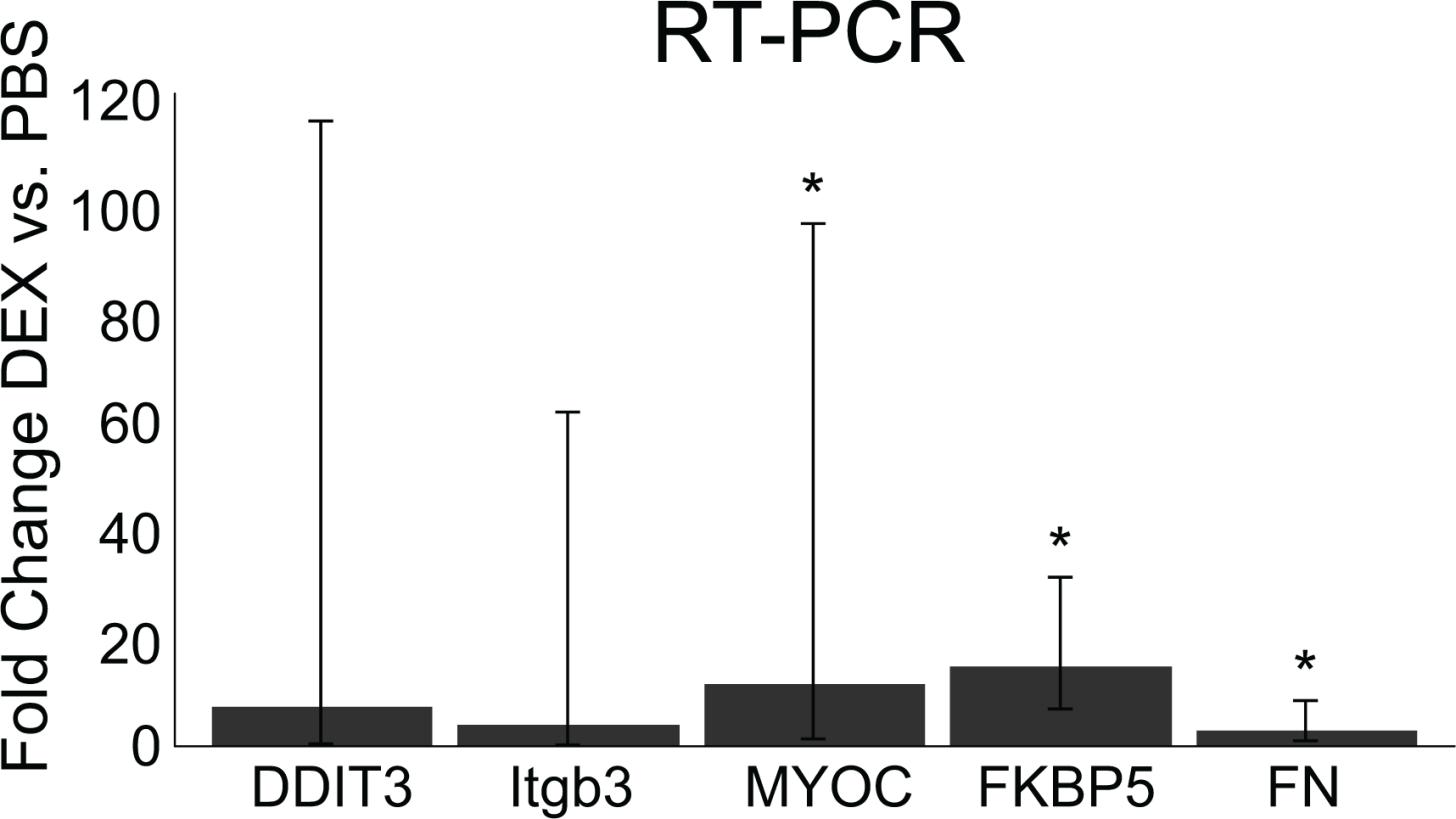
RT-PCR of RNA isolated from anterior segments of mice eyes treated with DEX or PBS for 3 weeks. Data are fold changes of DEX vs. PBS treatment after normalizing to the housekeeping gene SDHA. A fold change of 1 = no difference between DEX vs. PBS. Error bars represent lower and upper confidence levels. RNA levels significantly higher in DEX treated eyes, *p<0.05.

Lastly, we performed RT-PCR using some of the anterior segments of mice from the 3 week treatment group. As shown in Fig 8, there was no change in β3 integrin or CHOP mRNA levels when comparing DEX treated versus PBS treated eyes. We did detect significant increases in FKBP5 (14.6 fold; p<0.05), FN (2.91 fold; p<0.05) and MYOC (11.4 fold; p<0.05) mRNA in DEX treated eyes compared to PBS treated eyes. This suggests that DEX may have activated gene expression of some genes associated with the primary and secondary glucocorticoid responses after 3 weeks of treatment. However, this did not result in an increase in expression at the protein level (Fig 5A). This supports an earlier report by Bermudez et al [23] which suggested FN levels may be a secondary response in DEX treated TM cells. In addition, they found while mRNA levels often correlate with protein levels, this is not always the case, as we show here [41].

## Discussion

Our results showed that in mice which did not respond to DEX treatment with a prolonged elevation in IOP, there was a noticeable lack of the secondary glucocorticoid response. Neither β3 integrin nor MYOC, which are associated with the secondary glucocorticoid response, were upregulated in DEX treated eyes at either the 3 week time point where IOP was increased or at the 5 week time point where the IOP had returned to baseline. In addition, FN which also lacks GREs and thus can be considered part of the secondary glucocorticoid response was not statistically altered. This suggests that in mice which would be considered”steroid non-responders” because they did not demonstrate a sustained increase in IOP, there was failure to elicit a secondary glucocorticoid response. Clearly, it cannot be due to the responsiveness of the mice to DEX since the DEX treatment was effective. There was an increase in FKBP5 expression that resulted from a primary glucocorticoid response and the mice exhibited weight loss which is consistent with systemic effects of DEX. Also there was a short lived increase in IOP following the DEX treatment at 3 weeks. Thus the data suggest that in mice there may be a compensatory mechanism that can prevent or turn down the secondary glucocorticoid response to DEX. To the best of our knowledge this is the first attempt to study the secondary glucocorticoid response in vivo in association with IOP.

Interestingly, mice who responded to steroids with prolonged elevation in IOP showed an induction in the secondary glucocorticoid response [14, 15]. This suggests that the secondary glucocorticoid response may be responsible in part for the chronic elevation in IOP following steroid treatments. Similar to other studies [14, 16] we saw ~3mmHg increase in IOP with DEX treatment, although it was only transient in our mice. Why some C57BL/6 mice respond and others do not is unknown. It is possible that it could be the effectiveness of the delivery method. In the Overby et al. study [14], DEX was administered through a subcutaneous osmotic mini-pump implanted in the back, which could have resulted in a more effective and higher systemic DEX concentration. However, even with this technique the IOPs of some of their DEX treated mice fell within the range of their control mice. Zode et al. [15] gave eye drops similar to our method but they treated both eyes with DEX and we only treated one eye again raising the possibility that systemic levels were higher, although mouse weight and a systemic effect were not discussed in this paper. They also reported that 5–10% of their mice did not respond to the DEX eye drops with increased IOP. Interestingly, although we both used C57BL/6 strain of mice, their mice were bred in-house whereas ours were bought directly from Jackson Labs raising the possibility that differences in their mice response to DEX and others could be due to genetic differences in the various C57BL/6J mouse colonies.

To the best of our knowledge, this is the first time a weight loss has been reported in mice receiving topical eye drops of DEX. We attributed the weight loss in our DEX-treated mice to a systemic effect of the DEX since it is consistent with the systemic effect observed by Overby et al. [14] in mice who had DEX delivered via an implanted pump. The volume of the DEX drops used in our study (~30μl) most likely contributed to the systemic effects seen in our mice and enabled them to ingest the excess fluid not absorbed by the eye. Interestingly, despite this systemic effect we still did not see the sustained increase in IOP as seen in other studies [14–16], again supporting our conclusions that our mice exhibited a compensatory mechanism to the DEX induced increase in IOP.

Out of the 5 cohorts of mice we used for this study, one cohort of mice (Group 5) had abnormally high baseline IOPs. The normal range reported for C57BL/6J mice is between 12–18 mmHg [14, 15, 42–44]. This group of mice which were brought in at a later date than the first 4 cohorts had baseline IOPs ranging from 19–25 mmHg. Although it is not known why these mice had a higher baseline IOP, IOP can be affected by environmental factors such as the bedding, diet, light cycle, humidity and noise level of the housing and procedure rooms [43]. These environmental factors can also affect how rodents respond to anesthesia[43], which might affect IOP. Qiu et al. [45] published that ketamine can cause in increase in IOP in C57BL/6J mice within the first 5 minutes after anesthesia administration. Since the treatment of this particular group of mice was done after there were environmental changes in the bedding and diet in our animal facility, it is possible that one or all of these factors may have had an influence on IOP. Despite the high baseline IOPs, however, this cohort of DEX-treated mice still exhibited the same trend. There was an initial increase in IOP after 3 weeks of DEX treatment when compared to the PBS treated mice that returned to baseline by 5 weeks.

Several papers have shown that sensitivity to glucocorticoids may be dependent on polymorphisms in the glucocorticoid receptor (GR) gene (*NR3C1*)[46] or the ratio of the expression levels of the alternatively spliced isoforms GRα vs. GRβ [18, 47–50]. Unfortunately, we were unable to look at expression levels of GRα or GRβ in our mice because of the limited samples available for analysis. However, it is unlikely the ratios were different among our mice since the mice we used were an inbred strain. Furthermore, the fact that our mice showed an elevation of FKBP5 in response to DEX suggested there was an initial response to DEX and that the alternative spliced isoforms of GRβ may not be a factor.

A recent study by Bermudez et al. [23] supports our findings that a secondary glucocorticoid response is not always observed. In this study gene expression profiles of TM cells isolated from DEX responder or non-responder bovine organ cultured anterior segments were compared. As in our study they found that the cultured non-responder cell strains treated with DEX did not show an increase in FN expression, while the responders did. Furthermore, they were unable to detect any induction of MYOC which is also an indication the secondary glucocorticoid response was not activated. Thus, the lack of an increase in FN and MYOC expression in the non-responders support the idea that the “non-responders” fail to elicit the secondary glucocorticoid response and there may be a compensatory mechanism that controls the secondary glucocorticoid response.

Induction of MYOC and β3 integrin expression during the secondary glucocorticoid response in human TM cells is dependent on the phosphorylation of NFAT and its nuclear translocation via activation of the calcineurin/NFAT pathway (Fig 3) [19, 24]. A number of mechanisms have been reported to regulate NFAT nuclear translocation. For instance, polymorphisms in NFATc1 associated with congenital heart disease [51] may lead to impaired nuclear translocation and DNA binding affinity [52]. Translocation of cytoplasmic NFAT to the nucleus is also negatively repressed by a large complex of proteins that includes the noncoding repressor of NFAT (NRON) RNA molecule [53]. One of the proteins found in this NRON-protein complex called LRRK2 has recently been shown to contain polymorphisms that appear to alter or “fine tune” inhibition of NFAT translocation [54]. Expression of LRRK2 is also transactivated by the GR receptor and upregulated at both the transcriptional and translational levels [55]. Thus, depending on the functional activity of LRRK2 which can vary widely throughout the population due to its polymorphisms or NFATc1 polymorphisms, some individuals with certain alleles would exhibit a more robust response to glucocorticoids than others. Whether this complex which inhibits translocation independent of NFAT phosphorylation is responsible for regulating the secondary glucocorticoid response in TM cells is unknown. Gene array analysis has shown that LRRK2 is found in human TM cells and is upregulated 3.5 fold in DEX treated human TM cell cultures (the data can be downloaded from ProteomeCommons.org Tranche using the following hash: DzOJRE0nJjl7HokcNP3oq3iBcaPkLnMRCkjukv87GRvIYnN0nCCn-ZHpNuyvYjGEi9iOO[56]) suggesting that this protein would be present in DEX treated TM cells.

It was interesting that we saw a decrease in CHOP expression in the anterior segments at 3 weeks in DEX-treated mice. Recent studies have shown that in DEX-treated mice exhibiting an elevation in IOP, CHOP expression is upregulated and deletion of CHOP prevented the DEX induced ocular hypertension [15]. The failure to upregulate CHOP expression in this study might also partially explain why our mice did not develop a prolonged elevation in IOP. The mechanism used by the mice TM to prevent or downregulate the secondary glucocorticoid response would have prevented the over expression of proteins and activation of the UPR pathway which in turn causes an increase in CHOP and ER stress reported by Zode et. al. [15].

In summary, mice appear to have a compensatory mechanism that can prevent the secondary glucocorticoid response from occurring. Whether the secondary glucocorticoid response is responsible for the long term and chronic elevation of IOP in SIG remains to be determined. Clearly, more experiments need to be done to determine what role the secondary glucocorticoid response may play in the development of glaucoma and how it is regulated.

## Supporting information

S1 Fig CHOP expression after 5 weeks of DEX or PBS. No CHOP was detected in the treated, control or contralateral eyes, although it was detected after 3 weeks of DEX as indicated in Fig 7. CHOP ~ 27kDa. MW = molecular weight marker.
(EPS)

S2 Fig MYOC labeling of mouse eye treated with DEX for 5 weeks. (A) MYOC sera labeling shown in red (kindly provided by S.I. Tomorav, Mol. Bio. Cell. 2001; 21(22):7707–7713). The level of MYOC in the TM (red rectangle) is not higher than the rabbit non-immune sera shown in (B) indicating no MYOC labeling. Nuclei labeling is in blue. Each micrograph is two images merged together to show the entire iridocorneal angle and was taken using the same exposure times. Tissue processing and antibody labeling was done as described in Materials and methods. MYOC antibody was diluted 1:50. CM = ciliary muscle, R = retina. Similar results were seen with a commercial rabbit anti-MYOC antibody (Abcam cat #ab41552). Scale bar = 20 microns.
(EPS)
